# Does learner handover bias ratings, entrustment decisions, and feedback across repeated encounters with the same resident?

**DOI:** 10.1007/s10459-025-10460-5

**Published:** 2025-08-14

**Authors:** S. Humphrey-Murto, Julie D’Aoust, Samantha Halman, Tammy Shaw, Vijay J. Daniels, Lynfa Stroud, Irene Ma, Beth-Ann Cummings, Timothy J. Wood

**Affiliations:** 1 University of Ottawa, Ottawa, Canada; 2 University of Alberta, Edmonton, Canada; 3 University of Toronto, Toronto, Canada; 4 University of Calgary, Calgary, Canada; 5 McGill University, Montreal, Canada

**Keywords:** Bias, Rater cognition, Learner handover, Entrustment, Feedback, Assimilation effect

## Abstract

Learner Handover (LH) involves sharing information about learners between faculty supervisors, aligning with a growth mindset. Previous studies, however, demonstrate LH can bias subsequent ratings. Most of these studies collect ratings after a single encounter but faculty often have multiple interactions with learners potentially mitigating LH-related bias. This study explored if LH influences faculty ratings, entrustment decisions and feedback after observing several encounters of the same learner. Internal medicine faculty (*n* = 57) from five medical schools were randomly assigned to one of three study groups. Each group received either positive, negative or no LH prior to watching five simulated resident-patient encounter videos of the same white male resident. Participants rated each video using an entrustment scale, the Mini-CEX and provided written feedback. Feedback was assigned a valence score (-3 to + 3). There were no statistically significant differences between the mean ratings across the LH conditions (positive, control, negative) for entrustment [3.42, 3.26, 3.62], Mini-CEX [6.00, 5.90, 6.28] or feedback valence ratings [-0.34, -0.99, -0.74]. In the post-study questionnaire, most raters reported the LH had minimal effect on their decisions. Only 29% of raters guessed the true purpose of the study. Unlike previous studies, LH had no effect on ratings, entrustment decisions, or feedback after one encounter, nor over subsequent encounters with the same resident. These findings suggest LH’s influence may vary and highlight the need for replication under different conditions, including diverse genders and equity-deserving groups, to identify factors that contribute to or mitigate bias.

**Supplementary Information:**

The online version contains supplementary material available at 10.1007/s10459-025-10460-5.

## Introduction

Learner education handover, or learner handover (LH) is the sharing of information about learners across educational stages between faculty supervisors. LH aligns well with the growth mindset of competency based medical education (CBME), in which learners progress towards competency (Holmboe et al., 2010). For example, with prior knowledge of the strengths and weaknesses of a learner, faculty’s teaching and feedback can be focused on where it is most needed. Reported benefits of LH include more tailored learning, as well as more accurate assessment and improved patient safety (Morgan et al., 2020; Warm et al., 2017).

There are potential benefits to LH but concerns have been raised (Kassam et al., 2019; Mims et al., 2017), including the potential for LH to bias future assessments leading to learner stigmatization (Humphrey-Murto et al., 2019). Rater biases due to prior knowledge have been demonstrated in many settings (Humphrey-Murto et al., 2019), including medical education. For example, one relevant bias is the assimilation effect (Humphrey-Murto et al., 2019). (Shaw et al., 2021) provided faculty supervisors with LH describing a weak resident and found that supervisors produced ratings that were lower than if they had been provided with information describing a very strong resident, after watching the same performances (Shaw et al., 2021). This assimilation bias was even seen when raters were provided with limited information, such as being told a resident is “above-average” or “below-average” (Seehusen et al., 2023). Other studies, however, have failed to demonstrate bias under similar experimental conditions (Dory et al., 2021). It is unclear what variables might be contributing to varying results.

Context effects, as described in the psychology literature, refer to situations where a judgement is influenced by prior information about the rate (Palmer & Gore, 2014). As noted above, the *assimilation effect* refers to the situation when the prior information causes judgments to be shifted toward the stimulus, whereas the *contrast effect* occurs when the prior information displaces the judgment away from the stimulus (Smither et al., 1988). There are many factors that might influence context effects and these moderators are referred to as b*oundary conditions.* Examples of boundary conditions include the lag time between receiving the prior information and rating the target performance, whether the prior information is positive or negative, or whether the target performance being evaluated is average or extremely positive or negative (Humphrey-Murto et al., 2021a, b). Another important consideration is whether the information is acquired *indirectly* by receiving information about the learner from another source such as a written description, previous reputation or more formal learner handover (i.e., learner handover), or *directly* by observing the same learner multiple times, as may occur over the course of an in-patient rotation. Research appears to suggest that assimilation often occurs with indirect prior performance information and contrast often occurs with direct prior information however exceptions certainly exist (Humphrey-Murto et al., 2021a, b).

Most studies to date have examined the effect of LH on one subsequent performance evaluation. In other words, experimental studies have demonstrated that receiving information prior to rating a single performance can lead to a bias (Humphrey-Murto et al., 2019; Shaw et al., 2021; Seehusen et al., 2023). Although in some clinical rotations a learner may only interact with a faculty member once, often learners work with a faculty member over several days or shifts and there is limited research on the influence of LH on rating multiple subsequent performances.

In one study, undergraduate psychology students evaluated brief written statements about hypothetical employees where favorable prior performance information was followed by nine poor performances and poor prior performance information was followed by nine favorable performances (Hanges et al., 1991). After the first performance, an assimilation effect was demonstrated, however ratings became more consistent with ratee’s actual behavior over time. No such studies have been reported in a medical education context.

There are two additional factors that are relevant to any discussion of biases due to LH: entrustment and feedback. Entrustability rating scales are increasingly used to measure a trainee’s trajectory to independent practice (Cate et al., 2016). While the concept of entrustment applied to workplace-based assessment has been shown to improve rating discrimination and inter-rater reliability, it is unclear how LH may influence future entrustment decisions (Crossley & Jolly, 2012). Faculty supervisors have previously expressed concern that learner handover could create a self-fulfilling prophecy in which residents perceived as weak were not entrusted thus excluded from some learning opportunities (Humphrey-Murto et al., 2021a, b). This was noted in a recent study where faculty described reducing their level of entrustment upon receiving negative information about a resident with whom they had never worked (Humphrey-Murto et al., 2021a, b).

With regards to feedback, proponents of LH state targeted feedback is one of the goals, especially in the narrative. Various studies show conflicting findings with some showing feedback concordant with ratings (Ginsburg et al., 2017), another showing discordant feedback highlighting weakness not identified by the ratings (Cook et al., 2016), and another demonstrating the narrative stemming from LH led to targeted feedback (Dory et al., 2021).

In sum, while there is support for implementing LH during transitions between faculty supervisors, concerns remain that prior information—particularly one supervisor’s perspective—may bias the incoming faculty member’s ratings, entrustment decisions, and feedback.

Given the limitations of previous studies, the purpose of this study was to answer the following question:

Does LH influence faculty ratings, entrustment decisions and feedback after observing several performances of the same learner in a medical education context?

## Methods

### This study received approval from the Ottawa health science network research ethics board (OHSN REB)

#### Participants

Participants included faculty members and final-year post-graduate trainees (PGY-4 and 5s) within the Departments of Medicine at the Universities of Ottawa, Alberta, Toronto, Calgary, and McGill who had at least one-year experience assessing trainees. All Department of Medicine faculty involved with supervising residents from each respective university were invited to participate were contacted via email. We obtained ethics approval from the Ottawa Health Network Research Ethics Board (OHSN-REB), and local ethics boards at each site.

#### Videos

We created 5 videos of resident-patient encounters (see Table 1 for details) because a prior study showed that the bias from LH was lost by the 4th encounter (Hanges et al., 1991). Our team has experience creating authentic clinical performance videos (Shaw et al., 2021). Due to the nature of the study, one resident (white male, current family medicine resident from the University of Ottawa) acted in all five videos. A white male was used to avoid other potential biases related to BIPOC (Black, Indigenous, and people of color). He was trained to portray an average performance as he interacted with and managed five different standardized patients who portrayed typical internal medicine case presentations in an emergency room or ward setting. Management included components of the history, physical exam and impression and plan. An average performance was determined by consensus by five research team members with extensive experience supervising and assessing residents in the workplace (SH, TS, VD, JD, SHM). The videos were edited and revised as required to achieve this. The target performance was selected as average as previous research has demonstrated that context effects are seen most consistently when the target performance is average or borderline (Humphrey-Murto et al., 2021a, b). The videos ranged from 7 to 11 min in length.

**Table 1:** Study design and cases

Group	Video 1*	2	3	4	5
A Negative LH	Management of a 42 y female with exertional shortness of breath due to congestive heart failure (treatment side effect breast cancer)	Management of a 62 yo male presenting with fever, chills and flank pain (urosepsis)	Management of a 50 yo male presenting with chest pain (myocardial infarction)	Management of a 34 yo female with a painful swollen knee (septic arthritis)	Management of a 65 yo female presenting with epigastric pain and anemia (upper gastrointestinal bleed)
B Positive LH					
C Control (no LH)					

* All videos involve the SAME average resident managing patients in the emergency room or on the ward

#### Sessions

Raters who agreed to participate were offered the choice of several dates and times to accommodate their schedules. We held 29 small group virtual sessions (each with one to four participants) between October 6th, 2022 and May 26th, 2023. Each session lasted 60–75 min, and was facilitated by a research assistant.

We used quasi-randomization to accommodate rater availability. Each small group of participants was randomized to one of three experimental groups (Positive LH, Negative LH or Control). Co-variates such as University site, participant specialty, and years of experience were considered during allocation. Gender was not considered. Within a single session, we only included participants from a single group (Positive, Negative or Control) to avoid contamination from potential questions or comments and we did not allow participants to interact with each other.

Positive LH group participants received positive LH (describing strong prior performance) and then scored 5 average performances from a single resident in different clinical contexts. Negative group participants received negative LH (describing weak prior performance) followed by rating the same 5 average performances from the same resident. Details of the LH can be found in Appendix 1. The control group received no LH, and served as a control group. Performances were viewed in the same order in all sessions to control for order effect.

Participants were instructed to assess the clinical performance as they would normally in the emergency room or on the ward. The LH was provided once (as written comments on the slide), just before the first video was viewed. Performances were rated, and written feedback provided immediately after each video using first an entrustment-anchored scale (Appendix 2), followed by the mini-Clinical Evaluation Exercise (Mini-CEX) tool (Appendix 3).

After viewing and rating all videos, the participants completed an exit questionnaire which asked questions regarding participant demographics, perceived credibility of the information provided and explored what they thought the true purpose of the study was. The survey contained both select response items and free text responses (Appendix 4).

To blind participants to the true nature of the study, participants were told the research team was studying the use of the entrustment scale and Mini-CEX in the workplace.

#### Learner Handover (LH) form

Since there is currently no widely accepted LH protocol available, co-investigators (SHM, TS) created a LH form that summarizes resident skills in the competencies of medical expert, communicator/collaborator, and professionalism similar to that used in a previous study (Appendix 1) (Shaw et al., 2021). We scripted LH positive LH to describe a resident who excelled in several areas and negative LH to describe a resident who has several areas requiring attention. As a manipulation check, clinicians on the team reviewed the comments for authenticity (SH, JD, VD, LS).

#### Main outcome measures

The Entrustment Scale in this study was modelled after one of the Competence-by-Design (CBD) observation templates used by the Royal College of Physicians and Surgeons of Canada (RCPSC). It has been used in other performance-based assessment studies and can discriminate between learners at different levels of training across internal medicine residents (Halman et al., 2020) (see Appendix 2).

The Mini-CEX tool (Appendix 3) includes six 9-point rating scales representing different competencies, and one 9-point scale representing overall clinical competence. It was chosen as it has been extensively studied and is familiar to the faculty. We removed the counselling competency as it was not relevant.

We also administered a short exit questionnaire (free-text responses) at the end of the session to ascertain raters’ impression of the credibility of the LH (Appendix 4). As formal LH is not the usual practice at many universities, the questionnaire was also used to determine whether participants were able to deduce the true purpose of the study.

#### Statistical analysis

##### Sample size calculation

Using the standard deviation of the control group from Shaw et al. (Shaw et al., 2021) (0.74) and assuming an alpha of 0.05 and power of 0.80, to find a statistically significant difference in mean Mini-CEX ratings of 0.68 we required 19 raters per group (total 57). We used 0.68 because that was the difference between positive and negative ratings in the Mini-CEX noted in the Shaw study (Shaw et al., 2021).

##### Rating scale analysis

For Entrustment ratings, a 3 × 5 mixed ANOVA was conducted with LH condition (Positive, Negative, Control) as a between subject variable and order (1 to 5) as a within subject variable. This type of analysis will allow us to measure both a change in ratings across videos but also detect differences between raters in each LH condition. Of specific interest will be the presence of a statistically significant interaction between LH condition and order because this would suggest ratings were influenced by the LH instructions but changed as the rater saw more performances. Subsequent comparisons were done on a video-by-video level to determine how many performances were needed before ratings in each LH condition were equivalent across conditions. Effect sizes were also calculated.

We performed an identical analysis for the Mini-CEX using both the average of the 5-item scale (interview, physical, humanistic/professional, clinical judgement, organization) and the single overall global assessment. We also examined the correlation between the Mini-CEX and Entrustment ratings overall and on each video.

#### Feedback analysis

Initially, the feedback text was divided into phrases. Each phrase was coded for valence (positive, negative, or neutral) by coders who were blinded to the group from which the comments came, and the initial coding content categories were drafted by SHM using content analysis (Hsieh & Shannon, 2005). Subsequently, four members of the team used the outline independently on three pages of feedback comments (each page included comments from several raters for the same video), then met to refine definitions and consensus of the interpretation of the content and valence. Thereafter two members (TS, SHM) of the team independently coded the feedback, and any discrepancies were discussed to reach consensus.

In addition, we provided a global valence rating to the entire text feedback for each resident-patient encounter (i.e., extremely positive + 3, neutral 0 to extremely negative − 3). This was to counter any loss of meaning that might occur from dividing the narrative into multiple phrases. Two members of the research team independently provided a global valence based on their clinical judgement. Any discrepancies were reviewed with a third member of the research team and their final rating based on consensus. Like the previous analyses, a 3 × 5 mixed ANOVA was conducted with LH condition (Positive, Negative, Control) as a between subject variable and order (1 to 5) as a within subject variable.

We then compared the valence of the three LH groups; Positive, Negative LH and Group C (Control). For each video by each LH group, the number of positive and number of negative comments were counted. These comments were then analyzed using a 3 × 2 mixed ANOVA with the LH condition (positive, control, negative) treated as a between subject variable and valence (positive vs. negative comments) as a within subject variable.

For the exit questionnaire, descriptive statistics and content analysis were used (Hsieh & Shannon, 2005). Two team members (TS and SHM) independently reviewed the free text responses and came to consensus on content though an iterative process.

## Results

Across 5 sites, 57 participants were recruited. Participant rater demographics can be seen in Table 2.

**Table 2:** Participant rater demographics (*n* = 57)

	Control group (*n* = 19)	Positive LH group (*n* = 19)	Negative LH group (*n* = 19)	Total
**Site**				
Calgary	5	3	3	**11**
Edmonton	0	5	4	**9**
Montreal	2	2	3	**7**
Ottawa	6	5	4	**15**
Toronto	6	4	5	**15**
**Specialty**				
General Internal Medicine	12	13	18	**43**
Other	7	7	3	17**
**Gender**				
Female	11	8	12	**31**
Male	7	11	7	**25**
Non binary or prefer not to say)	1	0	0	**1**
**Level**				
PGY4/5-fellow	1	1	3	**5**
Faculty	18	18	16	**52**
**Number of years supervising trainees**				
0–2	0	1	0	**1**
3–5	6	5	7	**18**
6–10	5	8	5	**18**
> 10	8	5	7	**20**

*(any of Cardiology, Critical care, Endocrinology, Gastroenterology, Geriatrics, Hematology, Infectious Diseases, Medical Oncology, Nephrology, Pulmonary Medicine, Rheumatology)

** 3 participants practiced as Generalists and subspecialists

### Entrustment ratings

As seen in Table 3, there was no main effect of LH condition [F(2,54) = 2.22, *P* =.12, η_p_^2^ = 0.08.] Thus, LH had no effect on entrustment ratings. Analysis by video did demonstrate a statistically significant difference in video 4 with a lower score in the control group (mean 2.79) compared to the negative condition (mean 3.47) *P* =.020, η_p_^2^ = 0.13.

When comparing videos to evaluate consistency of the resident performance, there was a main effect of video [F(4,216) = 28.5, *P* <.001, η_p_^2^ = 0.35] with a low mean score of 2.84 for video 2 and a high score of 3.91 for video 1 but the interaction between LH and video was not statistically significant. [F(8,216) = 1.13, *P* =.34, η_p_^2^ = 0.04.

**Table 3:** Entrustment: mean entrustment ratings for 5 videos in learner education handover (LH) conditions (maximum score 5)*

	Positive LH (*n* = 19) Means (SD)	Control (*n* = 19) Means (SD)	Negative LH (*n* = 19) Means (SD)	Mean all participants (*n* = 57)***
Video 1	3.84 (0.83)	3.89 (0.81)	4.00 (0.75)	3.91 (0.79)
Video 2	2.89 (0.66)	2.63 (0.68)	3.00 (0.75)	2.84 (0.70)
Video 3	3.74 (0.81)	3.74 (0.81)	3.79 (0.63)	3.75 (0.74)
Video 4	3.21 (0.79)	2.79** (0.63)	3.47** (0.77)	3.16 (0.77)
Video 5	3.42 (0.90)	3.26 (0.87)	3.84 (0.69)	3.51 (0.85)
Average of all videos	3.42 (0.52)	3.26 (0.57)	3.62 (0.48)	3.43 (0.54)

Ratings*

1 = I would need to do 2 = I would need to walk them through 3 = I would need to prompt from time to time 4 = I needed to be there just in case 5 = I would not need to be there

** statistically significant difference in video 4 with a lower score in the control group (mean 2.79) compared to the negative condition (mean 3.47) *P* =.020, η_p_^2^ = 0.13

*** When comparing videos, there was a main effect of video (*P* <.001)

### Mini-CEX ratings

The Pearson correlations between the mean Mini-CEX score and overall clinical competence ratings were high for all videos (lowest correlation for video 4 was 0.89) hence we only report mean of the 5 -item scale.

**Fig. 1:**
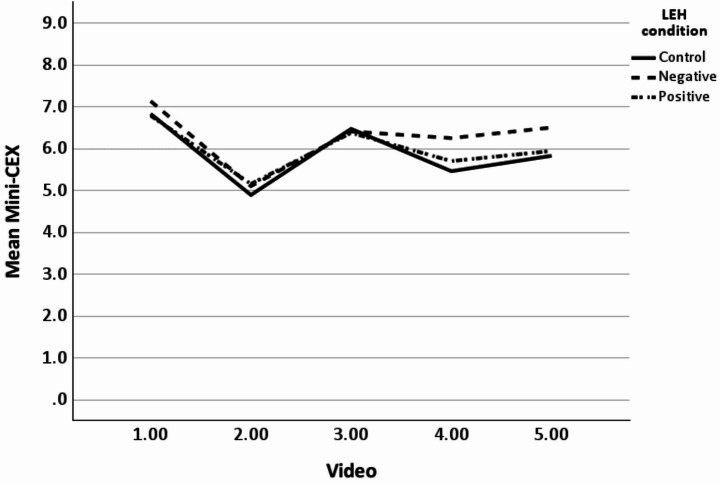
Mini-CEX ratings across 5 videos by Learner Handover Condition

The mean ratings for each video across the three conditions are displayed in Table 4. There were no statistically significant differences for the learner handover condition [F(2,54) = 0.81, *P* =.45, η_p_^2^ = 0.03], LH (Fig. 1). Analysis by video did demonstrate a statistically significant difference in video 4 with a lower score in the control group (mean 5.46) compared to the negative condition (mean 6.25) *P* =.04 η_p_^2^ = 0.11.

A significant main effect of video was noted for the Mini-CEX [F(4,216) = 47.74, *P* <.001, η_p_^2^ = 0.47. Mean Mini-CEX ratings for the videos ranged from a low of 5.05/9 for Video 2, to a maximum score of 6.92/9 for Video 1. The interaction between LH and video was not statistically significant. [F(8,216) = 1.25 *P* =.27, η_p_^2^ = 0.04)] Similar findings were observed for overall clinical competence.

The correlation between the Mini CEX) and the entrustment scale ratings for each video were; r (56) = 0.58 (video 1), 0.72 (video 2), 0.55 (video 3), 0.49 (video 4) and 0.53 (video 5) (*P* <.001).

**Table 4:** Mini-CEX: mean Mini-CEX ratings and overall competence score for 5 videos in learner education handover conditions (maximum score 9)*

	Positive LH (*n* = 19) Means (SD)	Control (*n* = 19) Means (SD)	Negative LH (*n* = 19) Means (SD)	Mean of mean Mini-CEX ratings*** (*n* = 57)	Overall clinical competence score (*n* = 57)
Video 1	6.79 (1.22)	6.83 (0.93)	7.14 (0.95	6.92 (1.04)	6.91 (1.33)
Video 2	5.16 (1.39)	4.89 (1.24)	5.10 (1.11	5.05 (1.23)	4.82 (1.23)
Video 3	6.38 (1.22)	6.47 (1.12)	6.41 (1.10	6.42 (1.13)	6.33 (1.20)
Video 4	5.70 (1.15)	5.46 (1.02)**	6.25 (1.34)**	5.81 (1.20)	5.28 (1.41)
Video 5	5.95 (1.40)	5.83 (1.16)	6.50 (1.25	6.10 (1.29)	5.84 (1.50)
Average of all 5 videos	6.00 (1.07)	5.90 (0.84)	6.28 (0.98)	6.06 (0.96)	5.84 (1.07)

LH: learner education handover

*Ratings 1–3: unsatisfactory; 4–6: satisfactory; 7–9: superior

** Statistically significant difference in video 4: control group (mean 5.46) compared to the negative condition (mean 6.25) *P* =.04

*** Significant main effect of video was noted for the Mini-CEX mean *P* <.001

### Feedback comments

There were no statistically significant differences in feedback global valence ratings for the learner handover condition [F(2,53) = 1.64 *P* =.20 η_p_^2^ = 0.06], LH (Table 5). A subsequent analysis by video revealed that only one video (video 1) had a main effect of LH condition [F(2,53) = 3.20, *P* =.05, η_p_^2^ = 0.11), with mean positive ratings higher in the positive condition compared to the other two conditions (*P* =.05 for both comparisons) but no difference in the feedback valence between the negative and control conditions (*P* =.58).

There was a statistically significant effect for video [F(4,212) = 25.71 P = < 0.001 η_p_^2^ = 0.33] with the mean rating for video 2 and 4 being lower than the other videos. The interaction between video and feedback was not statistically significant F(8,212) = 1.18, *P* =.31 η_p_^2^ = 0.04].

With regards to the number of comments, there was a statistically significant difference in the number of positive (M = 1.13 comments) and negative comments [M = 5.57 comments; F(1,53) = 132.34, *p* <.001, η_p_^2^ = 0.71) but there was no difference between LH conditions nor was the interaction statistically significant (Table 5).

Regarding feedback content, overall there was no difference between the number of comments between conditions. For only one video (video 5), there was a difference between groups with more communication comments in the control condition than the negative condition (0.58 vs. 0.00, *P* =.04) (Details in Appendix 5).

**Table 5:** Feedback Valence global ratings, number of positive and negative comments for each video in three learner handover conditions

Video	Mean Global Valence Rating (-3 to + 3)				Mean number of positive comments				Mean number of negative comments			
	LH condition				LH condition				LH condition			
	POS	CTRL	NEG	MEAN	POS	CTRL	NEG	MEAN	POS	CTRL	NEG	MEAN
1	0.79 **	-0.53	-0.22	0.02	3.21	2.53	2.39	2.71	1.95	3.42	2.33	2.57
2	-1.42	-1.79	-1.89	-1.70*	1.05	1.11	1.22	1.13*	5.47	5.47	5.78	5.57*
3	0.26	-0.21	-0.33	-0.09	2.79	2.47	2.11	2.46	2.84	2.95	3.00	2.93
4	-1.11	-1.58	-1.00	-1.23*	2.00	1.47	2.11	1.86	4.00	4.26	4.06	4.11 *
5	-0.21	-0.84	-0.28	-0.45	2.32	2.32	2.44	2.36	2.42	3.05	2.72	2.73
Average	-0.34	-0.99	-0.74	-0.69	2.27	1.98	2.05	2.10	3.34	3.83	3.58	3.58

* Main effect of video (*P* =.00, ƞƿ² = 0.327) Mean rating for video 2 lower than all other videos (*P* =.01)

** Mean global ratings higher in positive LH condition compared to control/negative (*P* =.05, ƞƿ² = 0.11) for video 1 only

### Exit questionnaire data

Exit questionnaire data showed that 23/38 (60.5%) felt the LH was credible, 9/38 (23.7%) felt it was not, 3 (7.8%) were unsure and 3 (7.8%) did not provide a clear response. Reasons for stating the information was credible included that it came from the program director, from multiple sources and consistent with what was observed. The main reason those that felt it was not credible reported was because it was not consistent with what was observed. Of participants who felt the LH was not credible 2 were from the positive LHLH and 4 from the negative LH condition. Only 29% (11/38) of raters in a LH condition guessed the true purpose of the study. To assess potential bias, raters were asked if they recognized the resident actor, who was a current family medicine trainee. Only 1 of 57 raters reported recognition.

## Discussion

This study explored the impact of LH on faculty ratings, entrustment decisions and narrative feedback after raters observed several performances of the same learner. Previous work has demonstrated that ratings were biased in the same direction as the information provided, otherwise known as an assimilation effect, but only after one observed encounter.

In our study, there was no difference between faculty ratings after multiple, or even the first single encounter. This pattern is contrary to previous work which demonstrated that ratings were biased in the same direction as the LH (Shaw et al., 2021, Seehusen et al., 2023). Other studies, however, have not demonstrated a difference in ratings (Dory et al., 2021). Why might results vary? All studies were randomized controlled trials with raters blinded to the true purpose of the study. In the Dory study, the LH described minor weaknesses in either communication or medical expertise, whereas the Shaw study described a resident who was excellent or very weak in all domains. Previous work outside the medical education context has suggested that the intensity of the prior information provided to a rater was important with very positive prior information having a greater effect than less positive information on subsequent ratings (Humphrey-Murto et al., 2021a, b). In the current study, the LH was very polarized across all domains, thus the lack of difference in ratings after the first encounter was not expected.

Another boundary condition relevant to our study is the level of performance of the target performance to be rated. It has been demonstrated that target performances that are ambiguous or average are particularly prone to the effects of prior information sharing (Humphrey-Murto et al., 2021a, b). In our study, we therefore attempted to make all performances “average”. However, it is clear the ratings varied from high for video 1 and a low for video 2 for entrustment and Mini-CEX ratings. Therefore, if raters saw a particularly strong performance, followed by an average performance they may have rated the second average performance lower than if they had not seen it immediately after a strong performance. This contrast effect has previously been noted where ratings of average performances after viewing 3 strong performances were lower than if viewed after 3 weak performances (Yeates et al., 2012) and in our study may have overshadowed the impact of the LH.

Another reason there was no difference between the groups may be that raters guessed the true purpose of our study, thus adjusting their ratings accordingly. Only 29% of rates guessed to true purpose of our study, vs 78% of rates in Shaw et al making guessing the purpose of the study an unlikely driver”.

In other studies participants are generally unaware of their susceptibility to the contextual manipulation (Yeates et al., 2012). Credibility of the information source may also play a role. In our study, 60% of raters felt the information was credible, however a third considered the information not credible, or of uncertain value.

Finally, the resident in this study was a white male. A white male was selected to reduce other potential factors that could influence a bias. For example, Lucey et al. (2020) demonstrated a bias based on race but Yeates et al. (2017) did not. Also, how raters combine information is unknown. Most studies have focused on examining biases in isolation, so it is unclear how raters use or consider multiple sources of data. The additive model proposes that raters combine data in an averaging fashion, to influence performance ratings (Hanges et al., 1991; Nieminen et al., 2013). In Nieminen’s study, the combined effect of prior information (positive, negative or none) and a ratee’s bodyweight (normal BMI or 40% above normal) on performance judgments was examined (Nieminen et al., 2013). The target performance was a videotape of a manager dealing with problem subordinate. As expected, the indirect prior information led to an assimilation effect. When the two factors were considered, the results demonstrated an additive model, such that past performance information and ratee’s bodyweight had co-occurring independent effects on the rating outcome (Nieminen et al., 2013). In our study, if the resident had been from an equity deserving group this might have influenced the outcome. This is certainly an avenue for future studies.

We found that feedback valence and content were not significantly different between LH groups overall. It is interesting that the valence of feedback was different in video 1, with more positive comments in the positive LH condition aligning with expectations. In the absence of significant differences in entrustment ratings or Mini-CEX ratings the meaning of this is less clear. Content of feedback also did not vary. This is not surprising as the learner handover was strong or weak across all domains, not specifically highlighting any areas to target.

There are several limitations of this study. This research was conducted in an experimental setting and may have limited transferability to real-world contexts where multiple factors are at play. For example, because it was in an experimental setting, participants may have deliberately responded in a more analytical manner in an attempt to emphasize accuracy than they would have in a more real-world setting. In clinical practice, faculty supervisors often face interruptions and cognitive load, which can increase the likelihood that their judgments are influenced by prior opinions (Halman et al., 2020). Another consideration is in the real world, there is often interaction between the rater and the learner. Previous work during job interview studies revealed that if the rater had a favorable first impression this lead to confirmatory behavior and styles from the interviewer and also from the interviewee (Dougherty et al., 1994). Quasi randomization always runs the risk of inequalities in randomization. The resident in this study was a white male, and future studies should explore how this might impact LH. Finally the very strong first performance may have led to a contrast effect, and is an attestation to the challenge of scripting videos to portray a certain level of performance.

Another limitation is related to the lack of power to find a difference. When deriving the sample size calculation, we had assumed a relatively large effect size when comparing ratings from any two conditions. We felt a large sample size was justified based on both the Shaw et al. study (Shaw et al., 2021), which used a similar methodology and other studies that had used the Mini-CEX to find differences between groups (e.g., Hatala et al. 2006). Unfortunately, in our study, the difference between the means in the conditions was smaller than expected thus leading to the lack of power. Given our results, it would have required close to 200 raters / group to find a significant difference.

Where does all this leave us? It appears that prior information in the form of LH has the potential to create bias, but predicting such effects remains elusive. In this study providing raters with information about a learner did not influence subsequent Mini-CEX ratings, entrustment ratings or feedback valence. Future studies should explore other factors such as: how LH effects vary with longer intervals between LH and observation; the LH effect when raters are cognitively overloaded; familiarity with the resident prior to receiving the LH, the combined effects of prior information and BIPOC and try to disentangle the effects of indirect and direct prior information when the same learner is viewed multiple time (Humphrey-Murto et al., 2021a, b).

Ultimately, accurate prediction may be impossible, prompting the medical education community to reflect on the primary goal of learner handover and consider alternative approaches, such as co-created learning plans, as potential solutions (Romanova et al., 2024).

## Supplementary Information

Below is the link to the electronic supplementary material.

Supplementary Material 1

Supplementary Material 2

Supplementary Material 3

Supplementary Material 4

Supplementary Material 5

## Data Availability

No datasets were generated or analysed during the current study.
