# Supplementary material for: Does learner handover bias ratings, entrustment decisions, and feedback across repeated encounters with the same resident?
Source: Adv Health Sci Educ Theory Pract. 2025 Aug 14;31(2):683–98. doi: 10.1007/s10459-025-10460-5 (PMC13046604; doi:10.1007/s10459-025-10460-5)
Supplement: Supplementary file 1 — Supplementary Material 1 [file 10459_2025_10460_MOESM1_ESM.docx]

**Appendix 1: Learner Education Handover Tool**

The Program Director is now forwarding educational information about each resident based on previous evaluations (ITERs, written examinations, OSCEs) over the last 12 months. Here is the information about this resident for you to reflect upon.

**Learner education handover tool (negative)**

**Level PGY-2: Internal Medicine**

*Medical expert*: Knowledge and physical examination skills have been identified as weak for level of training. This has been noted in OSCE and clinical evaluations. Difficulty integrating findings into comprehensive management plan. Procedural skills not observed.

*Communication/collaboration*: This has been flagged as an area requiring some attention. The resident, at times, has not included all members of the multidisciplinary team in the care plan.

*Professionalism*: Professional demeanor overall, but occasional poor follow-up and late to complete all tasks.

**Learner education handover tool (positive)**

**Level PGY-2: Internal Medicine**

*Medical expert*: Knowledge, physical examination skills and management skills excellent for level of training. Previous evaluations rate the trainee as “Excellent” or “Outstanding” and performing well above his peers. Noted to be very proficient in technical skills.

*Communication/collaboration*: Noted to be a strong communicator by previous preceptors.

*Professionalism*: Professional demeanor noted at all times. No issues identified.
