# Supplementary material for: Does learner handover bias ratings, entrustment decisions, and feedback across repeated encounters with the same resident?
Source: Adv Health Sci Educ Theory Pract. 2025 Aug 14;31(2):683–98. doi: 10.1007/s10459-025-10460-5 (PMC13046604; doi:10.1007/s10459-025-10460-5)
Supplement: Supplementary file 2 — Supplementary Material 2 [file 10459_2025_10460_MOESM2_ESM.docx]

**Appendix 2: Entrustment Scale (as used by Halman)**^19^

In your opinion, if faced with a similar case in the clinical setting, how would you rate this candidate’s performance?

| I would need to do (1)* | I would need to talk them through (2)* | I would need to prompt from time to time (3)* | Would need to be there just in case (4)* | I would not need to be there (5)* |
| --- | --- | --- | --- | --- |
| *Would require complete hands on guidance* | *Would be able to perform tasks but would require constant direction* | *Would demonstrates some independence, but would require intermittent direction* | *Independent but unaware of risks and still requires supervision for safe practice* | *Complete independence, understands risks and performs safely, practice ready* |
