# Supplementary material for: Does learner handover bias ratings, entrustment decisions, and feedback across repeated encounters with the same resident?
Source: Adv Health Sci Educ Theory Pract. 2025 Aug 14;31(2):683–98. doi: 10.1007/s10459-025-10460-5 (PMC13046604; doi:10.1007/s10459-025-10460-5)
Supplement: Supplementary file 3 — Supplementary Material 3 [file 10459_2025_10460_MOESM3_ESM.docx]

**Appendix 3: Mini CEX Tool and Feedback Form**

Please assess the resident’s performance using the relevant areas from the Mini-CEX tool provided below.

| **A** | MEDICAL INTERVIEWING SKILLS | | | | | | | | |
| --- | --- | --- | --- | --- | --- | --- | --- | --- | --- |
|  | 1 | 2 | 3 | 4 | 5 | 6 | 7 | 8 | 9 |
|  | Unsatisfactory | | | Satisfactory | | | Superior | | |

| **B** | PHYSICAL EXAMINATION SKILLS | | | | | | | | |
| --- | --- | --- | --- | --- | --- | --- | --- | --- | --- |
|  | 1 | 2 | 3 | 4 | 5 | 6 | 7 | 8 | 9 |
|  | Unsatisfactory | | | Satisfactory | | | Superior | | |

| **C** | HUMANISTIC QUALITIES/PROFESSIONALISM | | | | | | | | |
| --- | --- | --- | --- | --- | --- | --- | --- | --- | --- |
|  | 1 | 2 | 3 | 4 | 5 | 6 | 7 | 8 | 9 |
|  | Unsatisfactory | | | Satisfactory | | | Superior | | |

| **D** | CLINICAL JUDGMENT | | | | | | | | |
| --- | --- | --- | --- | --- | --- | --- | --- | --- | --- |
|  | 1 | 2 | 3 | 4 | 5 | 6 | 7 | 8 | 9 |
|  | Unsatisfactory | | | Satisfactory | | | Superior | | |

| **E** | COUNSELLING SKILLS | | | | | | | | |
| --- | --- | --- | --- | --- | --- | --- | --- | --- | --- |
|  | 1 | 2 | 3 | 4 | 5 | 6 | 7 | 8 | 9 |
|  | Unsatisfactory | | | Satisfactory | | | Superior | | |

| **F** | ORGANIZATION/EFFICIENCY | | | | | | | | |
| --- | --- | --- | --- | --- | --- | --- | --- | --- | --- |
|  | 1 | 2 | 3 | 4 | 5 | 6 | 7 | 8 | 9 |
|  | Unsatisfactory | | | Satisfactory | | | Superior | | |

| **G** | OVERALL CLINICAL COMPETENCE | | | | | | | | |
| --- | --- | --- | --- | --- | --- | --- | --- | --- | --- |
|  | 1 | 2 | 3 | 4 | 5 | 6 | 7 | 8 | 9 |
|  | Unsatisfactory | | | Satisfactory | | | Superior | | |

What feedback would you give the resident? : __________________________________________________________________________

______________________________________________________________________________

______________________________________________________________________________________________________________________________________________________________________________________________________________________________________________________________________________________________________________________________________________________________________________________________________
