# Supplementary material for: Does learner handover bias ratings, entrustment decisions, and feedback across repeated encounters with the same resident?
Source: Adv Health Sci Educ Theory Pract. 2025 Aug 14;31(2):683–98. doi: 10.1007/s10459-025-10460-5 (PMC13046604; doi:10.1007/s10459-025-10460-5)
Supplement: Supplementary file 4 — Supplementary Material 4 [file 10459_2025_10460_MOESM4_ESM.docx]

**Appendix 4:. Exit Post-Video Review Questionnaire**

1. What is your specialty? Select all that apply.

- General Internal Medicine
- Cardiology
- Critical Care
- Endocrinology
- Gastroenterology
- Geriatrics
- Hematology
- Medical Oncology
- Nephrology
- Respirology
- Rheumatology
- Other: please specify __________________________

1. How many years have you been in practice?

- 0-2
- 3-5
- 6-10
- >10

1. How many years have you been assessing trainees?
   - 0-2
   - 3-5
   - 6-10
   - >10
2. What is your gender?

- Male
- Female
- Non-binary
- Prefer not to answer

The following 5 questions used a scale from 1 - No Influence 5 - Neutral 10 - Significant Influence

5. To what extent did the Program Director Abbreviated Summary influence your completion of the Entrustment scale?

6. To what extent did the Program Director Abbreviated Summary influence your completion of the Mini-CEX?

7. To what extent did the Program Director Abbreviated Summary influence your completion of the Feedback comments?

8. To what extent did the Program Director Abbreviated Summary influence judgement on the first video?

9. To what extent did the Program Director Abbreviated Summary influence your judgement on the last video?

The following questions provided space for free text responses:

10. What did you think was the purpose of this study?

11. You were provided with information about the resident. The sharing of educational information between faculty involved in a trainee’s education is called Learner Handover (LHLH), previously known as “Forward Feeding”. Please answer the following questions and elaborate on your answers. *[only participants in the LHLH conditions received Q 11*]

a. How did you consider the learner handover information as you watched the video?

b. How did you use the learner handover information to inform your assessment of the Mini-CEX, your feedback and the Entrustment Scale?

c. Do you think the learner handover influenced your entrustment decisions? Why or why not?

d. What information, if any, provided to you in the learner handover information was relevant to you in making assessment judgments?

e. Did you feel the information provided was credible? Why or why not?

f. How do you think the learner handover influenced you on the first video compared to later videos?

12. Did you recognize the trainee in the videos? Yes or no

If yes, on a scale of 1 (poor) to 10 (excellent), what was your impression of this resident prior to this session?

13. What is your general impression regarding learner handover
