# Supplementary material for: Does learner handover bias ratings, entrustment decisions, and feedback across repeated encounters with the same resident?
Source: Adv Health Sci Educ Theory Pract. 2025 Aug 14;31(2):683–98. doi: 10.1007/s10459-025-10460-5 (PMC13046604; doi:10.1007/s10459-025-10460-5)
Supplement: Supplementary file 5 — Supplementary Material 5 [file 10459_2025_10460_MOESM5_ESM.docx]

**Appendix 5: Feedback Content Categories for each video (mean number of comments)**

| Video | LHLH condition | Data  gather | Comm | Physical  exam | Organization | Humanistic | Clin Judgment | Vague |
| --- | --- | --- | --- | --- | --- | --- | --- | --- |
| 1 | Pos | 0.84 | 0.42 | 0.84 | 0.26 | 0.21 | 1.95 | 0.68 |
|  | C | 0.95 | 0.89 | 1.42 | 0.42 | 0.16 | 1.95 | 0.26 |
|  | Neg | 0.94 | 0.89 | 1.00 | 0.33 | 0.11 | 1.39 | 0.39 |
| 2 | Pos | 0.89 | 0.26 | 1.32 | 0.16 | 1.11 | 2.74 | 0.11 |
|  | C | 0.89 | 0.37 | 1.58 | 0.26 | 1.37 | 2.00 | 0.21 |
|  | Neg | 0.78 | 0.50 | 1.00 | 0.11 | 1.33 | 3.06 | 0.28 |
| 3 | Pos | 0.74 | 0.37 | 0.79 | 0.05 | 0.26 | 3.26 | 0.32 |
|  | C | 0.95 | 0.26 | 0.95 | 0.05 | 0.37 | 2.53 | 0.47 |
|  | Neg | 0.78 | 0.11 | 0.78 | 0.17 | 0.17 | 3.00 | 0.50 |
| 4 | Pos | 0.95 | 0.21 | 0.89 | 0.26 | 0.21 | 3.11 | 0.53 |
|  | C | 0.89 | 0.26 | 1.05 | 0.42 | 0.05 | 3.05 | 0.16 |
|  | Neg | 1.33 | 0.22 | 0.94 | 0.39 | 0.11 | 2.94 | 0.44 |
| 5 | Pos | 0.42 | 0.21 | 1.26 | 0.21 | 0.26 | 2.05 | 0.58 |
|  | C | 0.74 | 0.58 | 1.53 | 0.32 | 0.16 | 2.11 | 0.26 |
|  | Neg | 1.00 | 0.00 | 1.33 | 0.17 | 0.22 | 2.28 | 0.22 |
|  |  |  |  |  |  |  |  |  |
